# Effects of Acute and One-Week Supplementation with Montmorency Tart Cherry Powder on Food-Induced Uremic Response and Markers of Health: A Proof-of-Concept Study

**DOI:** 10.3390/nu16193391

**Published:** 2024-10-06

**Authors:** Drew E. Gonzalez, Jacob A. Kendra, Broderick L. Dickerson, Choongsung Yoo, Joungbo Ko, Kay McAngus, Victoria Martinez, Megan Leonard, Sarah E. Johnson, Dante Xing, Ryan J. Sowinski, Christopher J. Rasmussen, Richard B. Kreider

**Affiliations:** Exercise & Sport Nutrition Lab, Department of Kinesiology and Sports Management, Texas A&M University, College Station, TX 77843, USA; dg18@tamu.edu (D.E.G.); jkendra@tamu.edu (J.A.K.); dickersobl5@email.tamu.edu (B.L.D.); choongsungyoo@tamu.edu (C.Y.); joungboko10@tamu.edu (J.K.); kirsten.nottingham@cpcmed.org (K.M.); victoria.jenkins@tamu.edu (V.M.); meganleonard10@tamu.edu (M.L.); sjohnson2216@tamu.edu (S.E.J.); dantexing@tamu.edu (D.X.); rjs370@tamu.edu (R.J.S.); crasmussen@tamu.edu (C.J.R.)

**Keywords:** polyphenols, oxidative stress, gout, hyperuricemia, purines

## Abstract

Metabolic conditions, such as gout, can result from elevated uric acid (UA) levels. Consuming high-purine meals increases UA levels. Therefore, people with hyperuricemia typically must avoid ingesting such foods. Polyphenols have been shown to reduce uric acid levels and tart cherries (TCs) are a rich source of phenolic and anthocyanin compounds. This proof-of-concept study evaluated whether ingesting TCs with a purine-rich meal affects the uricemic response. **Methods:** A total of 25 adults (15 males and 10 females, 85.0 ± 17 kg, 40.6 ± 9 years, 29.1 ± 4.9 kg/m^2^) with elevated fasting UA levels (5.8 ± 1.3 mg/dL) donated a fasting blood sample. In a randomized, double-blind, crossover, placebo-controlled, counterbalanced manner, participants ingested capsules containing 960 mg of a placebo (PLA) or concentrated TC powder containing 20.7 mg of proanthocyanins with a serving of hot soup (10 g of carbohydrate, 2 g protein, and 1 g fat) containing 3 g of purines (1 g of adenosine 5′-monophosphate, 1 g of disodium 5′-guanylate, and 1 g of disodium 5′-inosinate). Blood samples were obtained at 0, 60, 120, 180, and 240 min after ingestion to assess changes in uric acid levels and pharmacokinetic profiles. Cell blood counts, a comprehensive metabolic panel, cytokines, inflammatory markers, and subjective side effects ratings were analyzed on baseline (0 min) and post-treatment (240 min) samples. Participants continued consuming two capsules/day of the assigned treatment for one week and then repeated the experiment. Participants observed a 14-day washout and then repeated the experiment while ingesting the alternate treatment. Data were analyzed using general linear model (GLM) statistics with repeated measures, pairwise comparisons, and percentage change from baseline with 95% confidence intervals (CIs). **Results:** No statistically significant interaction effects or differences between treatments were seen in uric acid levels or PK profiles. Analysis of percent changes from baseline revealed that TC ingestion reduced the blood glucose levels following the ingestion of the high-purine meal (−4.2% [−7.7, −0.7], *p* = 0017). Additionally, there was some evidence that TC ingestion attenuated the increase from baseline in IL-1β and IL-10 and increased INF-γ. No significant differences were seen in the remaining health markers or subjective side effects ratings. **Conclusions:** Acute and one-week TC supplementation did not affect the uricemic response to ingesting a high-purine meal in individuals with mildly elevated UA levels. However, there was some evidence that TC supplementation may blunt the glycemic response to ingesting a meal and influence some inflammatory cytokines. Registered clinical trial NCT04837274.

## 1. Introduction

Gout is a form of inflammatory arthritis that is characterized by uric acid (UA) crystallization in and around the joints, resulting in discomfort, swelling, and pain [1]. Excessive UA accumulation results in hyperuricemia (i.e., serum UA levels > 6 mg/dL in males and >7 mg/dL in females), a metabolic disorder often developed from high purine dietary intake [2]. Hyperuricemia is estimated to affect 32.5 million Americans (14.6% of the population), with this number rising globally [3]. Urate-lowering therapies are commonly prescribed for those with recurring gout. Yet, evidence suggests low adherence to these prescriptions [4,5], implicating the need for strategies, such as dietary supplementation, to mitigate inflammatory disorders and related comorbidities.

Cherries, cherry juice, and tart cherry (TC) extract have garnered interest in gout management, largely due to their ability to lower serum UA levels among healthy and obese individuals [6]. Zhang and colleagues [7] reported that cherry intake (i.e., 2 days of 1–3 servings of cherries) was associated with lower gout attack risk (35%). Montmorency TC (*Prunus cerasus* L.) provides a rich source of polyphenols, anthocyanins, and flavonols and exerts antioxidant and anti-inflammatory effects purported to improve cardiometabolic health [8,9,10]. Several clinical studies have reported TC can reduce blood pressure, inflammation, and all-cause mortality risk [11,12,13,14]. In a recent study, Hillman and Uhranowsky [15] reported that (1) a single ingested capsule of powdered TC reduced UA levels (8%) in healthy young adults, and (2) the powdered TC capsules were more effective in reducing serum UA than a TC juice blend [15]. Regarding inflammation, Martin and Coles [16] reported that overweight and obese adults who consumed TC juice for four weeks decreased C-reactive protein (CRP, −19.4%) and monocyte chemoattractant protein-1 (−6.8%). Another study reported that 12 weeks of TC citrate supplementation in males with gout decreased CRP by 42% and was associated with fewer gout flare-ups [17].

While these data show promise, limited work has addressed how TC affects the uricemic response to ingesting high-purine meals. Given the prevalence of high-purine foods and diets, understanding the impact of TC supplementation on UA levels and markers of health factors can shed light on potential treatment strategies to prevent gout. Therefore, this study aimed to determine if TC ingestion with a high-purine meal would reduce increases in serum UA. The secondary objective was to determine if TC supplementation would lower pro-inflammatory cytokine responses and/or improve markers of cardiometabolic health.

## 2. Methods

### 2.1. Research Design

**Figure 1:**
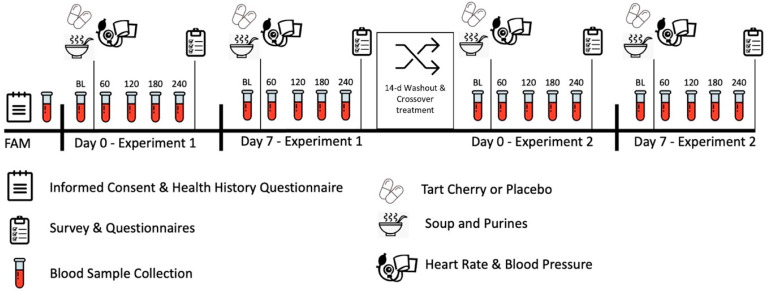
Experimental design and study timeline.

This trial was conducted in a randomized, double-blind, placebo-controlled, counterbalanced, and crossover manner with approval by the university Human Research Protection Program Internal Review Board (IRB2020-0164F, approved 4/10/2020) in accordance with the Declaration of Helsinki ethical principles for medical research involving human subjects. The study was also registered with clinicaltrials.gov (NCT04837274, submitted 3/31/21, posted 4/8/21). Individuals were recruited to partake in this proof-of-concept study assessing the impact of acute and one-week (i.e., 7 days) TC supplementation on food-induced elevations in serum UA, inflammatory cytokines, markers of cardiometabolic health, and self-reported side effects. The participants attended an acute and 7-day post-treatment laboratory session wherein fasted blood samples and self-reported side effects were collected pre-post a food-induced UA protocol. Participants observed a one-to-two-week washout and repeated the experiment with the alternative treatment. Figure 1 displays the general experimental design and testing sequences employed. The phlebotomy and blood analysis processes were performed in biological safety level 2 laboratories.

### 2.2. Study Participants

Participants were men and women between 30 and 60 years of age with fasted serum UA levels within the range of 5.5 and 8.0 mg/dL. Volunteers were excluded if they (1) were taking any dietary supplements or prescription medications known to influence UA; (2) had any known disease requiring regular prescription medication; (3) had any abnormal lab values from the screening blood sample; (4) had any known allergy to TC or immunoglobulin E; or (5) were pregnant, as well as trying to become pregnant or currently breastfeeding (women of childbearing potential were asked to take a urine pregnancy test to determine eligibility for the study). Volunteers were recruited using email, social media advertisements, and by posting study flyers. Interested participants underwent a phone screening to assess eligibility. Those meeting phone screening criteria were scheduled for a familiarization where they signed consent forms, completed health questionnaires, and donated a fasting blood sample to assess eligibility.

**Figure 2:**
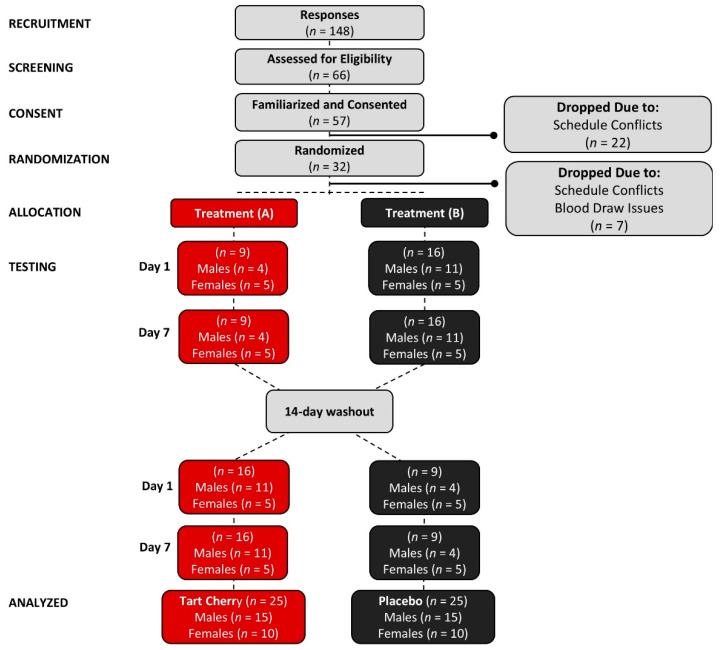
Consolidated Standards of Reporting Trials (CONSORT) chart. Unblinding revealed that the placebo treatment was Treatment B and tart cherry was Treatment A.

Figure 2 shows a Consolidated Standards of Reporting Trials (CONSORT) flow chart. A total of 148 people responded to study advertisements, 66 met the preliminary entrance criteria, 57 volunteers consented to participate, and 32 were randomized to start the study Seven participants withdrew because of issues with obtaining blood samples or scheduling conflicts. In all, 25 participants (10 women and 15 men with mildly elevated UA levels (5.8 ± 1.3 mg/dL) completed all study aspects.

### 2.3. Testing Sequence

Participants reported to the laboratory between 0500 and 1100 in a fasted state (≥12 h), returned a four-day dietary log, and answered side effects questionnaires. The participants rested seated for six minutes and then had their resting heart rate and blood pressure measured. Volunteers then had their height and weight measured and donated a blood sample. In a randomized manner, participants ingested their assigned treatment (either placebo or tart cherry) and then consumed soup containing purines. Blood samples were taken 60, 120, 180, and 240 after ingestion of the meal. Participants then completed a questionnaire about side effects.

### 2.4. Supplementation Protocol

Participants were randomly assigned to treatments using a Latin square-balanced design [18]. Participants ingested two 480 mg capsules (960 mg) containing rice-derived placebo (PLA, CeraLOK™, Irvine, CA, USA) or tart cherry powder (CherryPURE^®^, Shoreline Fruit LLC, Traverse, MI, USA) before ingesting a serving of dried soup (Lipton^®^ Soup Secrets^®^ Chicken Noodle Soup, Toronto, Nory York, Ontario, Canada) mixed in hot water. The soup contained 10 g of carbohydrate, 2 g of protein, and 1 g of fat with three grams of added purines (i.e., 1 g of adenosine 5′-monophosphate, 1 g of disodium 5′-guanylate, and 1 g of disodium 5′-inosinate, Salary Health (Wuxi) Co., Ltd., Wuxi, China). The TC powder consisted of Montmorency tart cherry powder derived from TC skins containing 2.16% proanthocyanins (20.7 mg per 960 mg serving). For comparison, Advanced Laboratories (Salt Lake City, UT, USA) conducted analytical testing and found that 31 mL (10.5 fl oz) of TC juice contains ≈600 mg and ≈40 mg of phenolic and anthocyanin compounds, respectively. Supplement contents were verified by Avanti Laboratories (Miami Lakes, FL, USA). Supplement capsules were matched for size and color and placed in labeled bottles for double-blind administration. Volunteers consumed two capsules per day of their assigned treatment for 7 days and then repeated the experiment. A 14-day washout was observed before repeating the experiment with the remaining treatment. Compliance was monitored via verbal communication, phone texts, and email.

## 3. Procedures

### 3.1. Diet Standardization

Volunteers were given a list of foods containing purines and asked to refrain from ingesting these foods for four days before testing. To ensure diet consistency throughout the study, volunteers recorded food and beverage consumption for four days before the first testing session using a food log or the MyFitnessPal Calorie Counter version 21.8.0 phone application (MyFitnessPal, Inc., Baltimore, MD, USA) [19]. The participants were asked to replicate their diet before each testing session. The dietary records were simply used to standardize the diet before testing sessions. Therefore, the dietary data were not analyzed.

### 3.2. Demographics

A calibrated Health-O-Meter Professional 500KL (Pelstar LLC, Alsip, IL, USA) scale was used to determine body weight and height. Resting heart rate and blood pressure were measured using standard procedures [20] with a calibrated Connex^®^ ProBP™ 3400 digital blood pressure device (Welch Allyn, Tilburg, The Netherlands).

### 3.3. Blood Collection and Analysis

Blood samples were collected in BD Vacutainer^®^ (Becton, Dickinson, and Company, Franklin Lakes, NJ, USA) serum separation (SST) and K2 ethylenediaminetetraacetic acid (EDTA) tubes by trained phlebotomists following standard procedures. After sitting for 15 min at room temperature, the SST tubes were centrifuged at 3500× *g* for 10 min in a refrigerated (4 °C) Thermo Scientific Heraeus MegaFuge 40R Centrifuge (Thermo Electron North America LLC, West Palm Beach, FL, USA) [4]. For subsequent analysis, serum samples were aliquoted and stored at −80 °C in microcentrifuge tubes (Eppendorf North America, Inc., Hauppauge, NY, USA). One SST and one EDTA tube were sent to Clinical Pathology Labs, Inc. (Austin, TX, USA, CLIA #45D0505003, CAP Accreditation #21525-01) for analysis using a Roche Cobas Gen 2 enzymatic/colorimetric analyzer (Roche Diagnostics International AG, Rotkreuz, Switzerland). The reported test repeatability CV for the primary outcome variable uric acid was 0.6% while remaining clinical chemistries ranged from 2% to 6%. A pharmacokinetic (PK) analysis was performed on UA levels using the PK Solution 2.0 Noncompartmental Pharmacokinetic Data Analysis software (Summit Research Services, Montrose, CO, USA). The UA levels and weight of participants observed following each testing session were entered into the PK software utilizing the single-dose, two-term analysis function. The software calculated the concentration maximum (Cmax), time to concentration maximum (Tmax), area under the curve (AUC), area under the moment curve (AUMC), and elimination and appearance/disappearance kinetics. Serum cytokine concentrations were measured using multiplex, magnetic bead-based Luminex™ MagPlex magnetic microsphere assays (Thermo Fisher Scientific, Waltham, MA, USA) with a Luminex MagPix instrument (Luminex Corporation, Austin, TX, USA. In our laboratory, the inter-assay and intra-assay variations for the serum cytokine assays range between 2 and 18% and 3 and 10% [21].

### 3.4. Side Effects Questionnaire

Subjective side effects were assessed using a previously published [21,22,23] categorical Likert scale. The test-to-test variability for this questionnaire has been reported to have a low CVs (1–3%) and moderate to high intraclass correlations (0.59 to 0.88) [21,22].

### 3.5. Statistical Analysis

The IBM SPSS^®^ 29.0 statistical software package (IBM Corp., Armonk, NY, USA) was used to analyze the results. An a priori sample size of 24 was deemed adequately powered in this crossover study based on our previous work [23,24,25], wherein we assume an expected 5% improvement and 80% power and recommendations on power determination for preliminary studies [26,27]. In addition, we have found that this sample size is sufficient for assessing clinical significance [28,29,30,31,32,33,34]. Sphericity was assessed using Mauchly’s test, while skewness and kurtosis statistics were used to assess distribution normality. A general linear model (GLM) with repeated measures for between-subjects (treatment) and within-subjects (time), as well as multivariate and univariate analysis, was performed to assess statistical hypotheses. The Wilks’ Lambda and Greenhouse–Geisser univariate correction tests assessed the interaction and time effects. Data were considered statistically significant when the probability of type I error was *p* < 0.05, while statistical tendencies (*p* > 0.05 to *p* < 0.10) are noted to reduce the probability of type II error [29,31]. Partial Eta squared (ηₚ^2^) statistics were used to assess effect size wherein effect sizes of 0.01 were small, 0.06 were medium, and 0.14 were considered large [35]. Fisher’s least significant difference statistics were used to assess post hoc and pairwise comparisons [36,37]. The clinical significance of the results was identified by assessing mean or percent mean changes from baseline with 95% confidence intervals (CI) [29,32,33]. Mean changes and 95% CI’s completely above or below the baseline were considered clinically significant. Chi-square analysis was used to analyze categorical questionnaire responses. Data are displayed as means with standard deviations (SDs) in tables and mean percent changes from baseline with 95% CIs (LL, UL) in figures. Adjusted series means [38], or the most common number for frequency data [39], were used to replace missing data (170 of 3650 data points; ≈5%).

## 4. Results

### 4.1. Demographic, Anthropometric, and Hemodynamic Data

The participants were 40.6 ± 8.9 years old, 171 ± 9 cm, 85 ± 17 kg, and 29 ± 5 kg/m^2^ with a resting heart rate of 69 ± 1.3 bpm, systolic blood pressure of 123 ± 1.8 mmHg, and diastolic blood pressure of 78 ± 1.0 mmHg. Table S1 presents hemodynamic and anthropometric data collected before each testing session. GLM analysis revealed no significant overall Wilk’s Lambda treatment x time effect (*p* = 0.969, 0.020), and no significant treatment x time effects were found in univariate analysis (*p* > 0.05).

### 4.2. Uric Acid

**Figure 3:**
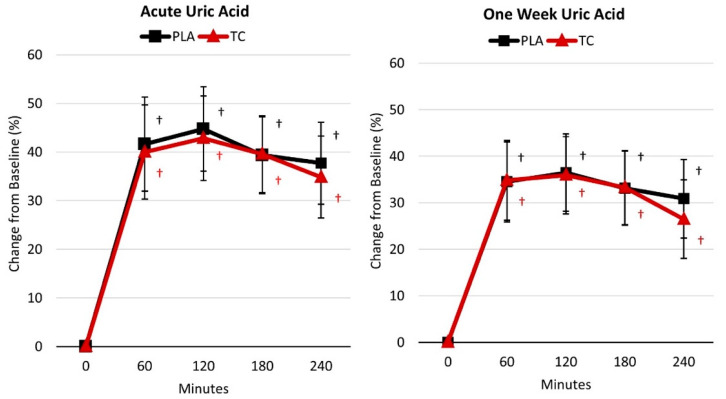
Percent changes in uric acid levels. PLA = placebo; TC = tart cherry; † = *p* < 0.05 difference from baseline.

Table S2 displays the uric acid response following acute and one-week supplementation. GLM analysis revealed no significant overall Wilk’s Lambda treatment x time effect (*p* = 0.928, 0.008), and univariate analysis found no treatment x time effects for the acute (*p* = 0.711, 0.008) nor one-week (*p* = 0.601, 0.011) supplementation duration. However, univariate analysis revealed significant time effects for the acute (*p* < 0.001, 0.764) and one-week (*p* < 0.001, 0.699) supplementation duration wherein uric acid levels were higher compared to baseline at all post-treatment time points (i.e., 60, 120, 180, and 240 min post-treatment) for both treatments. This can be seen in Figure 3, which presents the mean percent change from baseline with 95% CIs for the uric acid response for acute and one week of supplementation. Table S3 presents the plasma uric acid pharmacokinetic data. No significant treatment effects were found for any PK analysis variables for acute (*p* = 0.636, 0.110) and one-week (*p* = 0.805, 0.082) supplementation. Univariate analysis found no treatment effects for any PK analysis parameters (*p >* 0.05).

### 4.3. Inflammatory Cytokines

Table S4 displays the inflammatory cytokine response following acute and one-week supplementation. GLM analysis revealed no significant overall Wilk’s Lambda treatment x time effect (*p* = 0.409, 0.114) or significant time effect (*p* = 0.612, 0.100). Univariate analysis found no treatment x time effects or time effects for any inflammatory cytokines. When compared to baseline values within the respective supplementation protocol (i.e., acute versus one week), pairwise analysis revealed that interleukin-10 concentrations were lower 240 min post-treatment following the acute (*p* = 0.069) and one-week (*p* = 0.032) supplementation.

**Figure 4:**
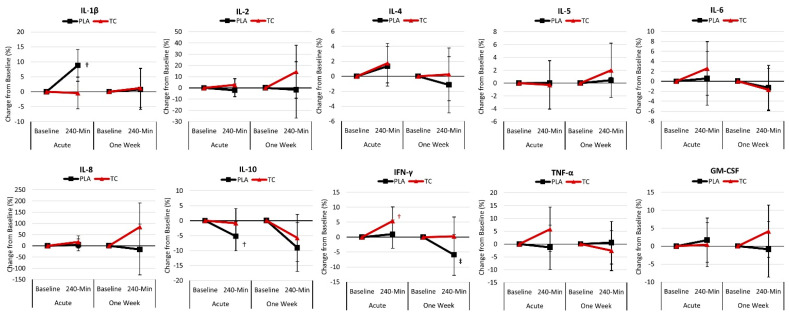
Percent change in inflammatory cytokines. PLA = placebo; TC = tart cherry; GM-CSF = granulocyte-macrophage colony-stimulating factor; IFN-γ = interferon-gamma; TNF-α = tumor necrosis factor-α; IL = inflammatory interleukins; † = *p* < 0.05 difference from baseline; ‡ = *p* > 0.05 to *p* < 0.10 difference from baseline.

Figure 4 presents the mean percent change from baseline with 95% CIs for the inflammatory cytokines. Regarding the acute supplementation, analysis of the percent mean change from baseline indicated that the inflammatory cytokines IL-1β (8.8% [3.5, 14.1], *p* = 0.002) increased above baseline with the placebo. In comparison, IFN-γ (5.3% [0.6, 10.1], *p* = 0.027) increased above baseline following the tart cherry treatment. In addition, IL-10 (−5.2% [−10.1, −0.3], *p* = 0.035) decreased below baseline following the placebo. Regarding the one-week supplementation, analysis of the percent mean change from baseline indicated that the inflammatory cytokines IL-10 (−9.0% [−17.4, −0.6], *p* = 0.035) decreased below baseline, while IFN-γ (−5.9% [−12.7, 0.9], *p* = 0.089) tended to decrease below baseline following the placebo.

### 4.4. General Health Markers

Tables S5–S8 present blood lipids, liver function biomarkers, whole red and white blood cell counts with percent differential, and serum glucose, electrolytes, and renal function biomarkers results following acute and one-week supplementation, respectively. GLM analysis found no significant treatment x time effects for blood lipids (*p* = 0.573, 0.044), liver function biomarkers (*p* = 0.529, 0.046), whole red and white blood cell counts with percent differential (*p* = 0.374, 0.109), and serum glucose, electrolytes, and renal function biomarkers (*p* = 0.803, 0.048) results. Some effects were observed over time; however, no significant univariate interaction effects were observed. All values were within normal clinical ranges.

**Figure 5:**
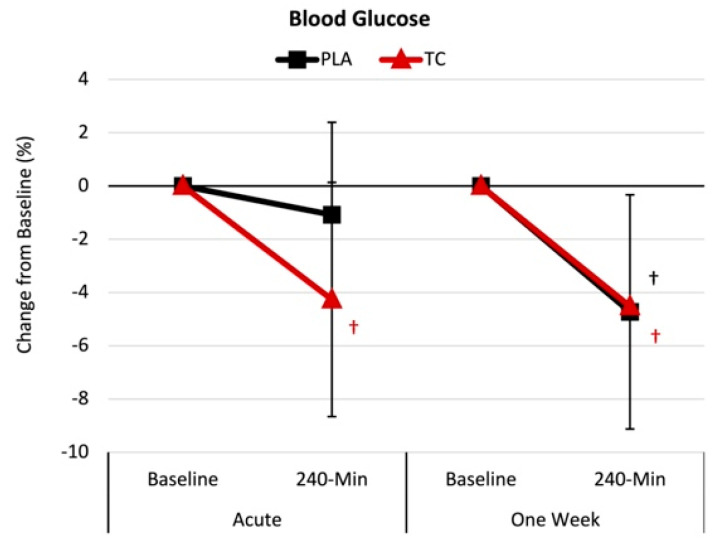
Blood glucose results. PLA = placebo; TC = tart cherry; † = *p* < 0.05 effect from baseline value.

Figure 5 presents the mean percent change from baseline with 95% CIs for blood glucose. Regarding the acute supplementation, analysis of the percent mean change from baseline indicated that blood glucose (−4.2% [−7.7, −0.7], *p* = 0.017) decreased below baseline following the tart cherry treatment but not the placebo. However, following the one-week supplementation, the tart cherry (−4.5% [−8.9, −0.1], *p* = 0.044) and placebo (−4.7% [−9.1, −0.3], *p* = 0.036) groups experienced reduced blood glucose.

### 4.5. Side Effects

Table S9 displays a Chi-square analysis of self-reported symptom frequency and severity ratings observed following acute and one-week supplementation. No differences were observed between the treatments regarding the self-reported frequency or severity of side effects.

## 5. Discussion

This proof-of-concept study examined whether acute and one-week powdered TC supplementation would lessen the effects of ingesting a purine-containing meal, lower pro-inflammatory cytokine responses, and/or improve general markers of cardiometabolic health. The main findings of this study indicate that acute and TC supplementation did not affect the uric acid or pharmacokinetic response to ingesting a purine-containing meal. However, there was evidence that TC ingestion lowered blood glucose levels 240 min following the meal. There was some evidence that TC ingestion attenuated the increase from baseline in IL-1β and IL-10 and increased INF-γ. However, no significant differences were seen in the remaining health markers or subjective side effects ratings. The following discussion provides additional insights into the results observed, study limitations, and future research recommendations.

### 5.1. Uric Acid Response

Acute and one-week powdered TC supplementation (960 mg) did not impact the uric acid levels in the blood after consuming a high-purine-containing meal among individuals with elevated serum UA levels. Only two other studies have assessed the impact of cherry consumption and the variations over time in UA responses [40,41]. Jacob and colleagues [40] assessed the impact of consuming two servings of cherries (280 g) among females aged 22–40 and found that plasma urate levels decreased (14.5%) 5 h post-consumption. Similarly, Bell et al. [41] assessed 30 and 60 mL of Montmorency TC concentrate on UA activity among healthy individuals and noted a 36% decrease from baseline serum urate levels following the 60 mL dose. Several other studies have shown favorable effects of cherry consumption on serum UA levels and gout flare-ups [7,16,42,43]. While these studies suggest cherries and TC can lessen UA activity over time, neither study assessed these responses after consuming a high-purine-containing meal. To our knowledge, this is the first study to evaluate the impact of ingesting TC prior to consuming a high-purine-containing meal. Considering the durations of previous work (i.e., 1 and 4 months) [3,43], a longer supplementation duration may be warranted to induce favorable effects on UA activity, especially following food-induced elevations in UA. Interestingly, chrysanthemum flower oil, which contains high polyphenol levels (similar to TC), has been assessed following diet-induced (i.e., high-purine-containing meal) serum UA elevation [44,45,46,47,48]. For example, Ueda and colleagues [44] demonstrated that chrysanthemum flower oil reduced the diet-induced serum UA response in individuals with baseline UA levels > 7.1 mg/dL. Another study by Hirano et al. [47] found that 4 weeks of 100 mg of luteolin-rich chrysanthemum flower extract reduced serum UA among individuals with baseline UA levels of 5.5 to 7.0 mg/dL. Lastly, Takara and colleagues [48] demonstrated that 100 mg of luteolin-rich chrysanthemum flower extract supplementation for 12 weeks lowered serum UA levels (−0.3 ± 0.7 mg/dL). Taken together, these findings suggest that high-polyphenol-containing compounds, such as TC, may need to be consumed for at least 4 weeks to reduce food-induced serum UA elevations.

### 5.2. Inflammatory Cytokine Response

We also assessed the impact of TC supplementation on the inflammatory cytokine response to ingesting a purine-containing meal. Except for the mean change from baseline values for IFN-γ (5.3% increase) following the acute TC supplementation, there was no effect on inflammatory cytokines. However, in the placebo treatment, IL-1β (8.8%) concentrations were elevated, while IL-10 (−5.2%) decreased, providing some evidence that TC supplementation may have blunted the inflammatory response following ingestion of a high-purine-containing meal. Contrary to our findings, previous work has demonstrated that consumption of cherries (whole food or supplementation sources) can reduce inflammatory cytokines [7,14,24,49,50,51]. For instance, Levers and colleagues [24] demonstrated that 480 mg/d for ten days of TC supplementation could lower inflammatory markers (47%) compared to placebo among 27 endurance-trained runners performing a half-marathon run time trial. However, participants supplemented their diet with TC for 14 days before performing the run, and the half marathon served as the stressor used to induce an inflammatory response [24]. The present study’s stressor was consuming a purine-containing meal, which is unlikely to elicit a similar response to running-induced muscle damage and inflammation. Cerletti et al. [52] also demonstrated that orange juice (containing 53.1 ± 5.3 mg/L of polyphonic and anthocyanin compounds) co-ingested with a fatty meal blunted the increase in WBC and myeloperoxidase degranulation, suggesting that this nutrition strategy may lessen the inflammatory response to ingesting a fatty meal. Certainly, a high-purine diet (i.e., the Western diet) can induce low-grade inflammation [53], fueling gout flare-ups [54]. However, the purine-containing meal used in the present study was a low-calorie serving of dried soup comprising only 10 g of carbohydrates, 2 g of protein, and 1 g of fat. It is plausible that a meal resembling a typical Western diet could induce a greater inflammatory response. Future research is warranted to examine the impact of TC supplementation following such a meal.

### 5.3. General Health Markers and Safety

We found that the acute TC supplementation reduced blood glucose levels (4.2%), whereas there was no change with the placebo. Following one week of supplementation, the TC (4.5%) and placebo (4.7%) treatments led to reductions in blood glucose levels. Several studies have assessed the impact of TC consumption on cardiometabolic risk factors (i.e., blood lipids, hemodynamics, etc.), wherein the most notable effect was reductions in blood glucose levels following four weeks of consuming 240 mL/d of cherry juice [55]. Desai and colleagues [13] observed that TC juice consumption (30 mL of TC concentrate diluted in 100 mL of water) for 6 days reduced fasting blood glucose (9%), suggesting TC’s potential ability to improve glycemic function within a cohort of insulin-resistant individuals. Another study by Desai et al. [12] demonstrated that acute TC supplementation (capsulated and juice forms) lowered insulin levels compared to placebo at 1 and 3 h, respectively. These studies underscore TC’s ability to impact individuals with metabolic syndrome favorably. Our study participants did not display criteria for metabolic syndrome and, apart from the elevated serum UA levels, were considered generally healthy. Our findings suggest that TC supplementation (960 mg) may improve glycemic function following a high-purine-containing meal. Future work is warranted to assess insulin and blood glucose to better understand TC’s ability to impact glycemic function following a high-purine-containing meal.

Other cardiometabolic risk factors have been assessed following cherry consumption and TC supplementation. A recent meta-analysis concluded that TC juice consumption did not affect blood pressure, triglycerides, total cholesterol, low-density lipoproteins (LDL), or high-density lipoproteins (HDL) levels [55]. Interestingly, Chai et al. [56] reported that consuming 480 mL/d of TC juice for 12 weeks lowered LDL (3%, 2 mg/dL) and systolic blood pressure (2.8%, ≈ 4 mmHg in older adults (65–80 years). Previous work has been carried out with other polyphenolic-rich compounds, such as beetroot juice [57] and blueberries [58], demonstrating systolic and diastolic blood pressure reductions. These authors have speculated that the reductions in blood pressure are likely due to increased nitric oxide production following their respective supplementation protocols [57,58]. Therefore, considering that TC, beetroot juice, and blueberries are similar in their high polyphenolic content, it has been proposed that TC would have a hypotensive effect on the host. However, we did not find an effect on resting hemodynamic measures following the acute and one-week TC supplementation protocol. In addition, we did not find any effect on blood lipids. Given that TC is rich in polyphenolic compounds, it has also been purported that TC could lessen oxidative stress and protect against lipid periodization of LDL. We did not assess oxidative stress markers within our present study; therefore, we cannot confirm if there was an antioxidant effect resulting from our supplementation protocol. Based on the previous work [56], it is plausible that a more extended duration supplementation protocol (i.e., 12 weeks) is needed to induce favorable effects for these cardiometabolic risk factors. Importantly, our results suggest that the TC supplementation did not adversely impact the general health markers or perceptions of side effects, suggesting that acute and one-week of TC supplementation was well tolerated.

### 5.4. Limitations

This proof-of-concept study is not without limitations. First, our study only evaluated six hours after ingesting a purine-containing meal. Since UA levels remained elevated, a longer assessment may be necessary to see if TC ingestion affects UA and/or PK profiles. Second, other than measuring UA, which is considered a general marker of oxidative stress [59], we did not assess the antioxidant effects of the TC supplementation, which are well documented [14,51,53,60]. Our proof-of-concept study aimed to assess TC’s ability to lower food-induced increases in serum UA levels. Future work is warranted to assess the potential antioxidant impact of TC following a high-purine-containing meal or food-induced elevations in serum UA. Second, we did not screen for metabolic syndrome, and considering other work relevant to this [12,13], individuals with high serum UA levels and metabolic syndrome may likely benefit from TC supplementation. Third, while the high-purine-containing meal did induce elevations in serum UA levels, a meal that more closely represents the Western diet is likely needed to trigger an inflammatory response. Future work is warranted to assess TC’s ability to blunt the inflammatory response to ingesting purine-containing meals and diets that resemble a typical Western meals and diet.

### 5.5. Conclusions

Acute and one-week powdered TC supplementation (960 mg containing 20.7 mg of proanthocyanins) did not promote statistically significant reductions in UA in response to ingestion of a high-purine-containing meal. However, TC ingestion decreased the glycemic response to the high-purine meal 240 min following the acute ingestion. There was some evidence that TC attenuated the increase from baseline in IL-1β and IL-10 and increased INF-γ. However, no significant differences were seen in the remaining health markers or subjective side effects ratings. Longer-duration studies are warranted to better understand the impact of TC on food-induced UA response and the anti-inflammatory and antioxidant effects.

## Data Availability

Data and statistical analyses are available for non-commercial scientific inquiry and/or educational use upon request to the corresponding author(s) as long as the use of data does not violate IRB restrictions, the sponsored research agreement, and the authors and sponsors of this work are appropriately acknowledged.

## Supplementary Materials

The following supporting information can be downloaded at: https://www.mdpi.com/article/10.3390/nu16193391/s1, Table S1: Hemodynamic and anthropometric data, Table S2: Uric acid response, Table S3: Plasma uric acid pharmacokinetic data ran on mean plasma values, Table S4: Inflammatory cytokine response, Table S5: Blood lipids, Table S6: Liver function biomarkers, Table S7: Whole blood cell counts with differential, Table S8: Glucose and renal function biomarkers, and Table S9: Frequency and severity of side effects following supplementation.
