# Supplementary material for: Effects of Acute and One-Week Supplementation with Montmorency Tart Cherry Powder on Food-Induced Uremic Response and Markers of Health: A Proof-of-Concept Study
Source: Nutrients. 2024 Oct 6;16(19):3391. doi: 10.3390/nu16193391 (PMC11478512; doi:10.3390/nu16193391)
Supplement: Supplementary file 1 [file nutrients-16-03391-s001.zip › Supplemental Tables R1.pdf]

Table S1. Hemodynamic and Anthropometic Data

Variable	Treatment	*n*	Acute	One Week		Effect	p-Value	η_p^2
Weight	PLA	*25*	84.99 ± 16.87	86.70 ± 21.11		T	0.273	0.025
(kg)	TC	*25*	85.11 ± 16.91	88.82 ± 21.21		G x T	0.682	0.004
Body Mass Index	PLA	*25*	29.08 ± 4.93	29.71 ± 7.24		T	0.319	0.021
(kg/m^2)	TC	*25*	29.11 ± 4.92	30.40 ± 7.56		G x T	0.732	0.002
Resting Heart Rate	PLA	*25*	122.88 ± 15.96	120.51 ± 12.15		T	0.729	0.003
(bpm)	TC	*25*	125.48 ± 14.92	121.56 ± 11.27		G x T	0.821	0.001
Systolic Blood Pressure	PLA	*25*	77.16 ± 9.41	75.79 ± 6.11		T	0.028	0.096
(mmHg)	TC	*25*	79.44 ± 8.82	77.82 ± 7.03	‡	G x T	0.579	0.006
Diastolic Blood Pressure	PLA	*25*	67.48 ± 10.99	67.64 ± 10.42		T	0.118	0.050
(mmHg)	TC	*25*	70.16 ± 11.09	70.93 ± 10.76		G x T	0.893	0.000

Data are expressed as means ± standard deviations for the placebo (PLA) and Tart Cherry (TC) treatments. Data were analyzed using a multivariate and univariate General Linear Model with repeated measures. P-levels, with partial ETA squared (ηp2) effect size, were reported. General Linear Model analysis revealed no significant overall Wilk's Lambda for Time ($p=0.087$, $\eta_p^2=0.212$) or significant Treatment x Time ($p=0.969$, $\eta_p^2 = 0.020$) effects. Greenhouse-Geisser univariate p-levels are listed for time (T) and treatment x time (G x T) interaction effects. Significance was determined via pairwise comparison, with LSD posthoc adjustment, indicated as differences from baseline values for acute and one week of supplementation: † = $p < 0.05$ [‡ = $p > 0.05$ to $p < 0.10$]. η_p^2 effect size values of 0.01 - 0.05 = small, 0.06 - 0.13 = medium, and >0.14 = large. HDL = High-Density Lipoprotein; LDL = Low-Density Lipoprotein; VLDL= Very Low-Density Lipoprotein

Table S2. Uric Acid Response

Variable		Treatment	*n*	0	60		120		180		240		Effect	p-Value	η_p^2
				Minutes											
Uric Acid	Acute	PLA	*25*	5.77 ± 1.18	8.13 ± 1.61	†	8.30 ± 1.51	†	8.00 ± 1.53	†	7.90 ± 1.48	†	T	<0.001	0.764
(mg/dL)		TC	*25*	5.92 ± 1.42	8.20 ± 1.54	†	8.32 ± 1.55	†	8.13 ± 1.41	†	7.80 ± 1.26	†	G x T	0.711	0.008
	One Week	PLA	*25*	5.78 ± 1.32	7.77 ± 1.71	†	7.86 ± 1.70	†	7.65 ± 1.63	†	7.52 ± 1.59	†	T	<0.001	0.699
		TC	*25*	6.10 ± 1.44	8.09 ± 1.41	†	8.19 ± 1.52	†	8.00 ± 1.44	†	7.58 ± 1.57	†	G x T	0.601	0.011

Data are expressed as means ± standard deviations for the placebo (PLA) and Tart Cherry (TC) treatments. Data were analyzed using a multivariate and univariate General Linear Model with repeated measures. P-levels, with partial ETA squared (ηp2) effect size, were reported. General Linear Model analysis revealed a significant overall Wilk's Lambda for Time ($p<0.001$, $\eta_p^2=0.574$); however, no significant Treatment x Time ($p=0.928$, $\eta_p^2 = 0.008$) effects. Greenhouse-Geisser univariate p-levels are listed for time (T) and treatment x time (G x T) interaction effects. Significance was determined via pairwise comparison, with LSD posthoc adjustment, indicated as differences from baseline: † = $p < 0.05$ [‡ = $p > 0.05$ to $p < 0.10$]. η_p^2 effect size values of 0.01 - 0.05 = small, 0.06 - 0.13 = medium, and >0.14 = large.

Table S3. Plasma Uric Acid Pharmacokinetic Data Ran on Mean Plasma Values.

Variable	Session	Group	N	Dosage	C max (obs)	N	Tmax	AUC (0-t) (obs area)	CL (obs area)	k21	E Phase Slope	E Phase Rate	D/A Phase Slope	D/A Phase Rate	D/A Phase k21	Effect	p-Level	η_p^2
				mg	mg/mL		hr	mg-hr/mL	mL/hr	1/hr	1/hr	1/hr	1/hr	1/hr	1/hr			
Uric Acid	*Acute*	PLA	*25*	11.76 ± 2.51	8.62 ± 1.63	*12*	2	31.68 ± 5.77	31.43 ± 6.66	0.271 ± 1.202	-0.005 ± 0.011	0.012 ± 0.025	-0.869 ± 2.933	2.002 ± 6.754	0.271 ± 1.202	Treatment	0.636	0.110
		TC	*25*	11.74 ± 2.50	8.52 ± 1.56	*11*	2	31.50 ± 5.52	31.42 ± 5.67	0.376 ± 1.098	-0.017 ± 0.034	0.040 ± 0.078	-1.476 ± 3.946	3.398 ± 9.087	0.376 ± 1.098			
		p-Value		0.983	0.832	χ^2	0.945	0.908	0.996	0.748	0.103	0.103	0.540	0.540	0.748			
	One Week	PLA	*25*	11.81 ± 2.50	8.08 ± 1.74	*9*	2	29.98 ± 6.37	33.75 ± 8.91	0.426 ± 0.752	-0.007 ± 0.012	0.016 ± 0.028	-0.356 ± 0.573	0.819 ± 1.319	0.426 ± 0.752	Treatment	0.805	0.082
		TC	*25*	11.76 ± 2.50	8.44 ± 1.50	*14*	2	31.12 ± 5.49	31.84 ± 6.01	0.362 ± 0.728	-0.026 ± 0.054	0.060 ± 0.125	-0.338 ± 0.573	0.778 ± 1.320	0.362 ± 0.728			
		p-Value		0.946	0.447	χ^2	0.232	0.501	0.380	0.760	0.095	0.095	0.914	0.914	0.760			

Data are expressed as means ± standard deviations for the placebo (PLA) and tart cherry (TC treatments. Tmax values are presented as time points with the highest frequency counts and significance shown with chi-squared (χ^2). Univariate and χ^2 p-values are listed under each variable. Differences between treatments are indicated by the following superscripts: † = p<0.05 and * = p>0.05 to p<0.10. Partial Eta squared effect sizes (ηp2) are reported as indicators of magnitude of effect where 0.01 was considered a small effect, 0.06 was considered a medium effect, and 0.14 was considered a large effect size. Cmax = Maximum observed concentration (from data). Tmax = Time at maximum observed concentration. AUC (0-t) = Cumulative area under curve for experimental time points only: AUC(0-t). CL (obs area) = Systemic clearance based on observed data points: E Phase Rate = Elimination Phase Rate. Rate = 2.303 x slope. E Phase Half-life = Time for concentration to diminish by one-half. Remains constant for 1st order kinetics. E Phase Slope = Elimination Phase Slope. Obtained by linear regression analysis of selected region on graph. D/A Phase Intercept = Distribution/Absorption phase intercept. For oral doses, the sign of the last coefficient (absorption phase) should be negative. D/A Phase Slope = Distribution/Absorption Phase Slope. Obtained by linear regression analysis of selected region on graph. D/A Phase Rate = Distribution/Absorption Phase Rate. Rate = 2.303 x slope. D/A Phase Half-life = Time or concentration to distribute/absorp by one-half. Remains constant for 1st order kinetics.

Table S4. Inflammatory Cytokine Response

Variable	Treatment	*n*	Acute Baseline	Acute 240-Min	One Week Baseline	One Week 240-Min	Effect	p-Value	η_p^2
IL-1β	PLA	*15*	0.27 ± 0.06	0.29 ± 0.03	0.28 ± 0.05	0.28 ± 0.05	T	0.67	0.02
(pg/mL)	TC	*17*	0.25 ± 0.03	0.25 ± 0.03	0.26 ± 0.05	0.26 ± 0.04	G x T	0.35	0.04
IL-2	PLA	*15*	10.91 ± 9.28	9.90 ± 7.15	11.91 ± 13.39	10.32 ± 8.68	T	0.65	0.01
(pg/mL)	TC	*17*	8.35 ± 5.44	8.25 ± 4.50	7.23 ± 3.71	7.83 ± 4.29	G x T	0.25	0.04
IL-4	PLA	*15*	7.99 ± 1.35	7.99 ± 1.35	8.22 ± 1.11	8.10 ± 1.09	T	0.83	0.01
(pg/mL)	TC	*17*	7.93 ± 0.69	8.05 ± 0.61	7.77 ± 0.51	7.77 ± 0.61	G x T	0.15	0.06
IL-5	PLA	*15*	0.57 ± 0.06	0.57 ± 0.07	0.57 ± 0.06	0.57 ± 0.06	T	0.38	0.03
(pg/mL)	TC	*17*	0.56 ± 0.05	0.57 ± 0.06	0.54 ± 0.05	0.55 ± 0.05	G x T	0.65	0.02
IL-6	PLA	*15*	8.95 ± 4.76	9.25 ± 5.77	9.00 ± 4.62	8.77 ± 4.29	T	0.12	0.07
(pg/mL)	TC	*17*	8.98 ± 5.55	9.60 ± 7.72	8.86 ± 6.01	8.78 ± 6.39	G x T	0.71	0.01
IL-8	PLA	*15*	11.07 ± 4.40	11.22 ± 5.50	12.76 ± 6.17	10.67 ± 6.29	T	0.40	0.03
(pg/mL)	TC	*17*	11.79 ± 4.44	13.29 ± 6.78	13.22 ± 6.36	19.82 ± 24.76	G x T	0.27	0.04
IL-10	PLA	*15*	1.42 ± 0.39	1.34 ± 0.39 ‡	1.46 ± 0.34	1.31 ± 0.30 †	T	0.19	0.05
(pg/mL)	TC	*17*	1.30 ± 0.34	1.28 ± 0.30	1.34 ± 0.34	1.29 ± 0.52	G x T	0.59	0.02
IFN-γ	PLA	*15*	1.41 ± 0.18	1.39 ± 0.22	1.41 ± 0.19	1.32 ± 0.17	T	0.44	0.03
(pg/mL)	TC	*17*	1.37 ± 0.32	1.42 ± 0.31	1.50 ± 0.45	1.48 ± 0.36	G x T	0.24	0.05
TNF-α	PLA	*15*	2.62 ± 1.46	2.68 ± 1.66	2.86 ± 1.98	2.70 ± 1.57	T	0.40	0.03
(pg/mL)	TC	*17*	2.71 ± 0.92	2.84 ± 0.97	2.92 ± 1.67	2.77 ± 1.45	G x T	0.91	0.00
GM-CSF	PLA	*15*	1.57 ± 0.51	1.65 ± 0.64	1.62 ± 0.52	1.60 ± 0.50	T	0.53	0.02
(pg/mL)	TC	*17*	1.44 ± 0.36	1.44 ± 0.36	1.47 ± 0.34	1.57 ± 0.65	G x T	0.45	0.03

Data are expressed as means ± standard deviations for the placebo (PLA) and Tart Cherry (TC) treatments. Note that the number of observations (n) may vary due to missing or inadequate samples: PLA (n=15) and TC (n=17). Data were analyzed using a multivariate and univariate General Linear Model with repeated measures. P-levels, with partial ETA squared (ηp2) effect size, were reported. General Linear Model analysis revealed no significant overall Wilk's Lambda for Time ($p=0.409$, η[illegible] =0.114) and no significant Treatment x Time ($p=0.612$, η[illegible] = 0.100) effects. Greenhouse-Geisser univariate p-levels are listed for time (T) and treatment x time (G x T) interaction effects. Significance was determined via pairwise comparison, with LSD posthoc adjustment, indicated as differences from baseline values for acute and one week of supplementation: † = $p < 0.05$ [‡ = $p > 0.05$ to $p < 0.10$]. η_p^2 effect size values of 0.01 - 0.05 = small, 0.06 - 0.13 = medium, and >0.14 = large. Interleukin 1β; IL-2 = Interleukin 2; IL-4 = Interleukin 4; IL-5 = Interleukin 5; IL-6 = Interleukin 6; IL-8 = Interleukin 8; IL-10 = Interleukin 10; GM-CSF = Granulocyte-macrophage colony-stimulating factor; IFN-γ = Interferon-gamma; TNF-α = tumor necrosis factor-alpha

Table S5. Blood Lipids

Variable	Treatment	*n*	Acute Baseline	Acute 240-Min		One Week Baseline	One Week 240-Min		Effect	p-Value	η_p^2
Cholesterol	PLA	*25*	190.16 ± 29.43	192.72 ± 31.09		189.52 ± 24.03	190.96 ± 23.87		T	0.949	0.001
(mg/dL)	TC	*25*	189.01 ± 25.28	185.36 ± 23.30	†	189.92 ± 30.67	188.01 ± 27.11		G x T	0.278	0.026
Triglycerides	PLA	*25*	147.12 ± 70.91	132.88 ± 68.32	†	142.28 ± 87.88	138.72 ± 81.91		T	0.110	0.046
(mg/dL)	TC	*25*	149.22 ± 80.44	132.68 ± 62.21	†	132.91 ± 72.25	127.16 ± 56.78		G x T	0.599	0.010
HDL Cholesterol	PLA	*25*	48.32 ± 17.58	49.04 ± 16.60		48.28 ± 15.17	49.24 ± 16.55		T	0.303	0.024
(mg/dL)	TC	*25*	47.03 ± 16.21	47.88 ± 16.23		47.76 ± 14.37	48.57 ± 14.52		G x T	0.829	0.003
LDL Cholesterol	PLA	*25*	115.96 ± 24.57	119.44 ± 26.19	†	116.72 ± 23.11	117.24 ± 22.94		T	0.840	0.003
(mg/dL)	TC	*25*	116.33 ± 25.41	113.56 ± 24.84	†	117.76 ± 26.68	115.73 ± 25.62		G x T	0.177	0.036
Non-HDL Cholesterol	PLA	*25*	141.84 ± 30.00	143.68 ± 32.01		141.24 ± 26.26	141.72 ± 26.37		T	0.728	0.006
(mg/dL)	TC	*25*	141.99 ± 29.03	137.48 ± 27.77	†	142.16 ± 31.05	139.43 ± 30.33		G x T	0.225	0.031
VLDL Cholesterol	PLA	*25*	25.88 ± 8.94	24.24 ± 8.90	‡	24.92 ± 9.60	24.48 ± 9.56		T	0.225	0.030
(mg/dL)	TC	*25*	24.70 ± 6.57	23.92 ± 7.73		24.40 ± 7.98	23.71 ± 7.70		G x T	0.884	0.003
LDL / HDL Cholesterol	PLA	*25*	2.71 ± 1.07	2.74 ± 1.10		2.68 ± 1.05	2.68 ± 1.09		T	0.329	0.022
Risk Ratio	TC	*25*	2.77 ± 1.18	2.69 ± 1.19	‡	2.69 ± 0.99	2.63 ± 1.02		G x T	0.565	0.010
Total Cholesterol / HDL Cholesterol	PLA	*25*	4.33 ± 1.34	4.30 ± 1.36		4.27 ± 1.26	4.24 ± 1.30		T	0.064	0.059
Risk Ratio	TC	*25*	4.42 ± 1.43	4.26 ± 1.39	†	4.27 ± 1.20	4.18 ± 1.23	¥	G x T	0.430	0.016

Data are expressed as means ± standard deviations for the placebo (PLA) and Tart Cherry (TC) treatments. Data were analyzed using a multivariate and univariate General Linear Model with repeated measures. P-levels, with partial ETA squared (ηp2) effect size, were reported. General Linear Model analysis revealed no significant overall Wilk's Lambda for Time (p=0.219, η_p^2 =0.059) nor significant Treatment x Time (p=0.573, η_p^2 = 0.044) effects. Greenhouse-Geisser univariate p-levels are listed for time (T) and treatment x time (G x T) interaction effects. Significance was determined via pairwise comparison, with LSD posthoc adjustment, indicated as differences from baseline values for acute († = p < 0.05 [‡ = p > 0.05 to p < 0.10]) and one week of supplementation (€= p < 0.05 [¥= p > 0.05 to p < 0.10]). η_p^2 effect size values of 0.01 - 0.05 = small, 0.06 - 0.13 = medium, and >0.14 = large. HDL = High-Density Lipoprotein; LDL = Low-Density Lipoprotein; VLDL= Very Low-Density Lipoprotein

Table S6. Liver Function Biomarkers

Variable	Treatment	*n*	Acute Baseline	Acute 240-Min		One Week Baseline	One Week 240-Min		Effect	p-Value	η_p^2
Total Protein	PLA	*25*	7.09 ± 0.44	7.15 ± 0.48		7.01 ± 0.38	7.18 ± 0.46	€	T	0.144	0.037
(g/dL)	TC	*25*	6.98 ± 0.33	6.97 ± 0.39		7.02 ± 0.41	7.05 ± 0.40		G x T	0.202	0.032
Albumin	PLA	*25*	4.48 ± 0.22	4.53 ± 0.25		4.44 ± 0.26	4.53 ± 0.25	€	T	0.117	0.041
(g/dL)	TC	*25*	4.46 ± 0.24	4.46 ± 0.24		4.45 ± 0.32	4.49 ± 0.23		G x T	0.632	0.012
Globulin	PLA	*25*	2.61 ± 0.36	2.62 ± 0.39		2.57 ± 0.35	2.65 ± 0.38	¥	T	0.489	0.016
(g/dL)	TC	*25*	2.53 ± 0.31	2.51 ± 0.35		2.57 ± 0.36	2.56 ± 0.34		G x T	0.264	0.027
Albumin / Globulin	PLA	*25*	1.76 ± 0.28	1.77 ± 0.29		1.77 ± 0.30	1.75 ± 0.27		T	0.586	0.013
Ratio	TC	*25*	1.80 ± 0.27	1.82 ± 0.32		1.76 ± 0.29	1.79 ± 0.27		G x T	0.546	0.014
Bilirubin	PLA	*25*	0.41 ± 0.21	0.45 ± 0.21	‡	0.45 ± 0.24	0.47 ± 0.22		T	0.039	0.064
(mg/dL)	TC	*25*	0.47 ± 0.29	0.46 ± 0.29		0.49 ± 0.27	0.53 ± 0.27		G x T	0.588	0.011
Alkaline Phosphatase	PLA	*25*	74.96 ± 16.15	74.12 ± 17.49		76.16 ± 17.28	75.00 ± 18.41		T	0.010	0.087
(u/L)	TC	*25*	76.32 ± 12.58	71.51 ± 13.30	†	74.28 ± 15.88	72.96 ± 14.78		G x T	0.106	0.045
Aspartate Aminotransferase	PLA	*25*	22.72 ± 6.74	23.52 ± 7.47		22.04 ± 7.50	22.12 ± 7.88		T	0.483	0.014
(u/L)	TC	*25*	22.61 ± 8.74	22.50 ± 8.65		25.28 ± 13.57	22.60 ± 8.27		G x T	0.176	0.037
Alanine Aminotransferase	PLA	*25*	27.40 ± 14.02	27.52 ± 14.16		25.08 ± 12.24	25.88 ± 13.39		T	0.375	0.019
(u/L)	TC	*25*	27.00 ± 14.42	26.64 ± 13.82		27.32 ± 15.17	26.50 ± 13.81		G x T	0.235	0.030

Data are expressed as means ± standard deviations for the placebo (PLA) and Tart Cherry (TC) treatments. Data were analyzed using a multivariate and univariate General Linear Model with repeated measures. P-levels, with partial ETA squared (ηp2) effect size, were reported. General Linear Model analysis revealed a significant overall Wilk's Lambda for Time (p<0.001, η_p^2 =0.117); however, there were no significant Treatment x Time (p=0.529, η_p^2 = 0.046) effects. Greenhouse-Geisser univariate p-levels are listed for time (T) and treatment x time (G x T) interaction effects. Significance was determined via pairwise comparison, with LSD posthoc adjustment, indicated as differences from baseline values for acute († = p < 0.05 [‡ = p > 0.05 to p < 0.10]) and one week of supplementation (€= p < 0.05 [¥= p > 0.05 to p < 0.10]). η_p^2 effect size values of 0.01 - 0.05 = small, 0.06 - 0.13 = medium, and >0.14 = large.

Table S7. Whole Blood Cell Counts With Differential

Variable	Treatment	*n*	Acute Baseline	Acute 240-Min		One Week Baseline	One Week 240-Min		Effect	p-Value	η_p^2
White Blood Cells	PLA	*25*	6.09 ± 1.66	5.79 ± 1.26		6.08 ± 1.52	5.76 ± 1.46	€	T	0.008	0.087
(k/uL)	TC	*25*	6.19 ± 1.51	5.98 ± 1.14		5.70 ± 1.18	5.62 ± 0.89		G x T	0.091	0.046
Red Blood Cells	PLA	*25*	4.65 ± 0.42	4.68 ± 0.47		4.64 ± 0.42	4.67 ± 0.44		T	0.162	0.038
(m/uL)	TC	*25*	4.67 ± 0.41	4.60 ± 0.44	‡	4.59 ± 0.40	4.43 ± 0.68	¥	G x T	0.089	0.051
Hemoglobin	PLA	*25*	13.98 ± 1.01	13.96 ± 1.10		13.91 ± 1.21	13.96 ± 1.10		T	0.149	0.038
(g/dL)	TC	*25*	14.00 ± 1.02	13.75 ± 1.14	†	13.72 ± 1.12	13.66 ± 1.13		G x T	0.297	0.025
Hematocrit	PLA	*25*	40.73 ± 2.81	40.75 ± 3.24		40.59 ± 3.06	40.75 ± 2.90		T	0.308	0.025
(%)	TC	*25*	40.54 ± 2.70	40.06 ± 2.99		39.92 ± 2.82	39.64 ± 3.09		G x T	0.340	0.023
Mean Corpuscular Volume	PLA	*25*	87.76 ± 4.11	87.45 ± 4.22		87.67 ± 4.02	87.46 ± 4.33		T	0.674	0.008
(fL)	TC	*25*	87.08 ± 3.91	87.77 ± 4.30	‡	87.22 ± 4.10	87.28 ± 4.17		G x T	0.203	0.033
Mean Corpuscular Hemoglobin	PLA	*25*	30.13 ± 1.87	29.94 ± 1.82		30.04 ± 1.84	29.96 ± 1.86		T	0.383	0.021
(pg)	TC	*25*	30.08 ± 1.72	29.99 ± 1.77		29.98 ± 1.71	30.08 ± 1.61		G x T	0.621	0.012
Mean Corpuscular Hemoglobin Concentration	PLA	*25*	34.32 ± 0.94	34.23 ± 0.85		34.26 ± 0.84	34.24 ± 0.77		T	0.515	0.016
(g/dL)	TC	*25*	34.52 ± 0.67	34.30 ± 0.83		34.34 ± 0.80	34.46 ± 0.76		G x T	0.856	0.005
Red Blood Cell Distribution Width	PLA	*25*	12.79 ± 0.55	12.76 ± 0.48		12.83 ± 0.54	12.74 ± 0.50	€	T	0.146	0.037
(%)	TC	*25*	12.77 ± 0.50	12.78 ± 0.48		12.84 ± 0.44	12.82 ± 0.52		G x T	0.479	0.017
Neutrophils	PLA	*25*	52.90 ± 7.21	54.14 ± 6.81		51.57 ± 9.00	53.46 ± 9.18	€	T	0.048	0.056
(%)	TC	*25*	51.81 ± 8.07	53.84 ± 8.48		51.58 ± 6.88	51.91 ± 6.56		G x T	0.759	0.007
Lymphocytes	PLA	*25*	34.17 ± 7.16	33.90 ± 6.89		35.34 ± 9.20	34.68 ± 9.26		T	0.323	0.024
(%)	TC	*25*	35.14 ± 7.87	34.09 ± 7.80		35.09 ± 6.86	35.63 ± 6.12		G x T	0.771	0.007
Neutrophils / Lymphocytes	PLA	*25*	1.65 ± 0.52	1.70 ± 0.57		1.65 ± 0.83	1.75 ± 0.92		T	0.518	0.015
Ratio	TC	*25*	1.61 ± 0.70	1.73 ± 0.70		1.55 ± 0.47	1.53 ± 0.45		G x T	0.419	0.019
Monocytes	PLA	*25*	8.36 ± 2.10	8.16 ± 2.03		8.55 ± 2.40	8.09 ± 2.06	€	T	0.393	0.020
(%)	TC	*25*	8.53 ± 2.08	8.35 ± 2.19		8.69 ± 2.43	8.56 ± 2.35		G x T	0.820	0.005
Eosinophils	PLA	*25*	3.49 ± 2.19	2.78 ± 1.70	†	3.48 ± 1.78	2.78 ± 1.61	€	T	0.000	0.267
(%)	TC	*25*	3.50 ± 1.92	2.84 ± 1.60	†	3.58 ± 1.95	2.95 ± 1.84	€	G x T	0.918	0.003
Basophils	PLA	*25*	0.79 ± 0.40	0.82 ± 0.38		0.89 ± 0.43	0.83 ± 0.36		T	0.272	0.027
(%)	TC	*25*	0.85 ± 0.38	0.78 ± 0.33		0.86 ± 0.45	0.81 ± 0.34		G x T	0.561	0.014
Platelet Counts	PLA	*25*	262.68 ± 64.58	267.64 ± 63.16		262.00 ± 62.20	263.60 ± 65.05		T	0.312	0.024
(k/uL)	TC	*25*	256.52 ± 54.71	258.56 ± 50.88		251.48 ± 60.57	251.72 ± 58.30		G x T	0.828	0.005

Data are expressed as means ± standard deviations for the placebo (PLA) and Tart Cherry (TC) treatments. Data were analyzed using a multivariate and univariate General Linear Model with repeated measures. P-levels, with partial ETA squared (ηp2) effect size, were reported. General Linear Model analysis revealed a significant overall Wilk's Lambda for Time ($p<0.001$, η_p^2 =0.218); however, there were no significant Treatment x Time ($p=0.374$, η_p^2 = 0.109) effects. Greenhouse-Geisser univariate p-levels are listed for time (T) and treatment x time (G x T) interaction effects. Significance was determined via pairwise comparison, with LSD posthoc adjustment, indicated as differences from baseline values for acute († = p < 0.05 [‡ = p > 0.05 to p < 0.10]) and one week of supplementation (€= p < 0.05 [¥= p > 0.05 to p < 0.10]). η_p^2 effect size values of 0.01 - 0.05 = small, 0.06 - 0.13 = medium, and >0.14 = large.

Table S8. Glucose and Renal Function Biomarkers

Variable	Treatment	*n*	Acute Baseline			Acute 240-Min				One Week Baseline			One Week 240-Min				Effect	p-Value	η_p^2
Glucose	PLA	*25*	98.36	±	10.90	96.56	±	5.91		98.28	±	9.21	93.04	±	5.36	€	T	0.002	0.109
(mg/dL)	TC	*25*	97.51	±	8.68	92.92	±	7.13	†	99.08	±	14.82	93.27	±	8.08	€	G x T	0.457	0.017
Creatinine	PLA	*25*	15.99	±	5.15	14.67	±	4.34	†	15.30	±	5.34	14.40	±	5.62		T	0.335	0.019
(mg/dL)	TC	*25*	16.42	±	3.49	16.08	±	3.62	†	15.94	±	5.34	15.30	±	3.94		G x T	0.327	0.020
Blood Urea Nitrogen	PLA	*25*	0.88	±	0.16	0.85	±	0.15	†	0.88	±	0.16	3.48	±	13.23	€	T	0.000	0.172
(mg/dL)	TC	*25*	0.88	±	0.17	0.82	±	0.15	†	0.87	±	0.14	0.83	±	0.15	€	G x T	0.806	0.004
Blood Urea Nitrogen/Creatinine Ratio	PLA	*25*	13.52	±	3.02	12.08	±	2.60	†	13.04	±	2.89	11.92	±	2.06		T	0.100	0.047
	TC	*25*	14.10	±	2.29	12.95	±	2.47		13.44	±	3.42	12.45	±	2.57		G x T	0.717	0.007
Sodium	PLA	*25*	139.64	±	2.14	141.08	±	2.14	†	140.20	±	2.36	140.64	±	2.48		T	0.013	0.075
(meq/L)	TC	*25*	139.86	±	2.39	140.39	±	1.89		139.92	±	1.96	140.76	±	2.01		G x T	0.527	0.015
Potassium	PLA	*25*	4.26	±	0.28	4.34	±	0.30		4.26	±	0.34	4.21	±	0.29		T	0.632	0.012
(meq/L)	TC	*25*	4.23	±	0.20	4.24	±	0.26		4.28	±	0.30	4.28	±	0.30		G x T	0.189	0.033
Chloride	PLA	*25*	102.64	±	2.51	104.08	±	2.12	†	103.40	±	2.10	103.52	±	1.85		T	0.012	0.078
(meq/L)	TC	*25*	103.00	±	2.22	103.87	±	2.28	†	103.64	±	1.91	103.69	±	2.19		G x T	0.828	0.005
Carbon Dioxide	PLA	*25*	24.24	±	1.74	24.56	±	2.27		23.88	±	2.52	25.16	±	1.75	€	T	0.011	0.0766
(meq/L)	TC	*25*	24.24	±	2.08	24.90	±	1.62	‡	24.52	±	2.47	24.98	±	1.40		G x T	0.515	0.0155
Calcium	PLA	*25*	9.36	±	0.33	9.28	±	0.36		9.42	±	0.29	9.26	±	0.37	€	T	0.001	0.1151
(mg/dL)	TC	*25*	9.34	±	0.26	9.11	±	0.30	†	9.34	±	0.33	9.23	±	0.35	¥	G x T	0.374	0.021

Data are expressed as means ± standard deviations for the placebo (PLA) and Tart Cherry (TC) treatments. Data were analyzed using a multivariate and univariate General Linear Model with repeated measures. P-levels, with partial ETA squared (ηp2) effect size, were reported. General Linear Model analysis revealed a significant overall Wilk's Lambda for Time ($p<0.001$, η_p^2 =0.190); however, there were no significant Treatment x Time ($p=0.803$, η_p^2 = 0.048) effects. Greenhouse-Geisser univariate p-levels are listed for time (T) and treatment x time (G x T) interaction effects. Significance was determined via pairwise comparison, with LSD posthoc adjustment, indicated as differences from baseline values for acute († = $p < 0.05$ [‡ = $p > 0.05$ to $p < 0.10$]) and one week of supplementation (€= $p < 0.05$ [¥= $p > 0.05$ to $p < 0.10$]). η_p^2 effect size values of 0.01 - 0.05 = small, 0.06 - 0.13 = medium, and >0.14 = large.

Table S9. Frequency and Severity of Side Effects Following Supplementation.

			Acute Ingestion														One-Week														
			Pre-ingestion (0-min)							Post-ingestion (240-min)							Pre-ingestion (0-min)							Post-ingestion (240-min)						
			Rating							Rating							Rating							Rating						
	**Symptom**	**Group**	**0**	**1**	**2**	**3**	**4**	**5**	**χ^2**	**0**	**1**	**2**	**3**	**4**	**5**	**χ^2**	**0**	**1**	**2**	**3**	**4**	**5**	**χ^2**	**0**	**1**	**2**	**3**	**4**	**5**	**χ^2**
**Frequency**	Dizziness	PLA	24	1	0	0	0	0	0.55	25	0	0	0	0	0	-	24	1	0	0	0	0	0.31	25	0	0	0	0	0	-
		TC	23	2	0	0	0	0		25	0	0	0	0	0		25	0	0	0	0	0		25	0	0	0	0	0	
	Headache	PLA	20	5	0	0	0	0	0.60	24	1	0	0	0	0	0.08	20	4	1	0	0	0	1.00	23	2	0	0	0	0	0.64
		TC	19	5	1	0	0	0		20	5	0	0	0	0		20	4	1	0	0	0		22	3	0	0	0	0	
	Tachycardia	PLA	25	0	0	0	0	0	-	25	0	0	0	0	0	-	25	0	0	0	0	0	-	25	0	0	0	0	0	-
		TC	25	0	0	0	0	0		25	0	0	0	0	0		25	0	0	0	0	0		25	0	0	0	0	0	
	Heart Palpitations	PLA	25	0	0	0	0	0	-	25	0	0	0	0	0	-	25	0	0	0	0	0	-	25	0	0	0	0	0	-
		TC	25	0	0	0	0	0		25	0	0	0	0	0		25	0	0	0	0	0		25	0	0	0	0	0	
	Dyspnea	PLA	24	1	0	0	0	0	1.00	25	0	0	0	0	0	-	24	1	0	0	0	0	0.31	25	0	0	0	0	0	-
		TC	24	1	0	0	0	0		25	0	0	0	0	0		25	0	0	0	0	0		25	0	0	0	0	0	
	Nervousness	PLA	22	3	0	0	0	0	0.53	24	1	0	0	0	0	0.55	22	2	1	0	0	0	0.84	24	1	0	0	0	0	1.00
		TC	21	2	1	1	0	0		23	2	0	0	0	0		23	1	1	0	0	0		24	1	0	0	0	0	
	Blurred Vision	PLA	25	0	0	0	0	0	-	25	0	0	0	0	0	-	25	0	0	0	0	0	-	25	0	0	0	0	0	-
		TC	25	0	0	0	0	0		25	0	0	0	0	0		25	0	0	0	0	0		25	0	0	0	0	0	
	Other	PLA	25	0	0	0	0	0	-	25	0	0	0	0	0	-	24	1	0	0	0	0	0.31	25	0	0	0	0	0	-
		TC	25	0	0	0	0	0		25	0	0	0	0	0		25	0	0	0	0	0		25	0	0	0	0	0	
**Severity**	Dizziness	PLA	24	1	0	0	0	0	1.00	25	0	0	0	0	0	0.31	24	1	0	0	0	0	0.31	25	0	0	0	0	0	-
		TC	24	1	0	0	0	0		24	1	0	0	0	0		25	0	0	0	0	0		25	0	0	0	0	0	
	Headache	PLA	20	4	0	1	0	0	0.54	23	2	0	0	0	0	0.27	22	3	0	0	0	0	0.54	24	1	0	0	0	0	0.30
		TC	19	3	2	1	0	0		18	5	1	1	0	0		20	4	1	0	0	0		22	3	0	0	0	0	
	Tachycardia	PLA	24	1	0	0	0	0	0.31	25	0	0	0	0	0	0.31	25	0	0	0	0	0	-	25	0	0	0	0	0	-
		TC	25	0	0	0	0	0		24	1	0	0	0	0		25	0	0	0	0	0		25	0	0	0	0	0	
	Heart Palpitations	PLA	25	0	0	0	0	0	-	25	0	0	0	0	0	0.31	25	0	0	0	0	0	-	25	0	0	0	0	0	-
		TC	25	0	0	0	0	0		24	1	0	0	0	0		25	0	0	0	0	0		25	0	0	0	0	0	
	Dyspnea	PLA	25	0	0	0	0	0	-	25	0	0	0	0	0	0.31	24	1	0	0	0	0	0.31	25	0	0	0	0	0	-
		TC	25	0	0	0	0	0		24	1	0	0	0	0		25	0	0	0	0	0		25	0	0	0	0	0	
	Nervousness	PLA	22	3	0	0	0	0	0.60	24	1	0	0	0	0	1.00	23	2	0	0	0	0	0.55	24	1	0	0	0	0	1.00
		TC	21	3	1	0	0	0		24	1	0	0	0	0		24	1	0	0	0	0		24	1	0	0	0	0	
	Blurred Vision	PLA	25	0	0	0	0	0	-	25	0	0	0	0	0	-	25	0	0	0	0	0	-	25	0	0	0	0	0	-
		TC	25	0	0	0	0	0		25	0	0	0	0	0		25	0	0	0	0	0		25	0	0	0	0	0	
	Other	PLA	25	0	0	0	0	0	-	25	0	0	0	0	0	-	24	0	1	0	0	0	0.37	25	0	0	0	0	0	-
		TC	25	0	0	0	0	0		25	0	0	0	0	0		24	1	0	0	0	0		25	0	0	0	0	0	

Data are presented as frequencies. PLA = Placebo; TC = Tart Cherry. Statistical significance is detailed by chi-squared analysis. Totals are included but, separate from analysis. Frequency rating scale: 0 = none; 1 = minimal (1-2 per/wk); 2 = slight (3-4 per/wk); 3 = moderate (5-6 per/wk); 4 = severe (7-8 per/wk); 5 = very severe (9 or more per/wk). Severity rating scale: 0 = none; 1 = minimal; 2 = slight; 3 = moderate; 4 = severe; 5 = very severe.
